# The role of pulmonary arterial stiffness in COPD

**DOI:** 10.1016/j.rmed.2015.06.005

**Published:** 2015-11

**Authors:** Jonathan R. Weir-McCall, Allan D. Struthers, Brian J. Lipworth, J. Graeme Houston

**Affiliations:** a Division of Cardiovascular and Diabetes Medicine, Medical Research Institute, University of Dundee, Dundee, United Kingdom; b Scottish Centre for Respiratory Research, Medical Research Institute, University of Dundee, Dundee, United Kingdom

**Keywords:** Pulmonary disease, Chronic obstructive, Hypertension, Pulmonary, Vascular capacitance, Vascular resistance, Pulmonary heart disease

## Abstract

COPD is the second most common cause of pulmonary hypertension, and is a common complication of severe COPD with significant implications for both quality of life and mortality. However, the use of a rigid diagnostic threshold of a mean pulmonary arterial pressure (mPAP) of ≥25mHg when considering the impact of the pulmonary vasculature on symptoms and disease is misleading. Even minimal exertion causes oxygen desaturation and elevations in mPAP, with right ventricular hypertrophy and dilatation present in patients with mild to moderate COPD with pressures below the threshold for diagnosis of pulmonary hypertension. This has significant implications, with right ventricular dysfunction associated with poorer exercise capability and increased mortality independent of pulmonary function tests.

The compliance of the pulmonary artery (PA) is a key component in decoupling the right ventricle from the pulmonary bed, allowing the right ventricle to work at maximum efficiency and protecting the microcirculation from large pressure gradients. PA stiffness increases with the severity of COPD, and correlates well with the presence of exercise induced pulmonary hypertension. A curvilinear relationship exists between PA distensibility and mPAP and pulmonary vascular resistance (PVR) with marked loss of distensibility before a rapid rise in mPAP and PVR occurs with resultant right ventricular failure. This combination of features suggests PA stiffness as a promising biomarker for early detection of pulmonary vascular disease, and to play a role in right ventricular failure in COPD. Early detection would open this up as a potential therapeutic target before end stage arterial remodelling occurs.

## Highlights

• Pulmonary hypertension is common in COPD.
• Right ventricular remodeling occurs at pressures below the diagnostic threshold of PH.
• Pulmonary arterial stiffening occurs early in the development of PH.
• Non-invasive measurement of pulmonary stiffness may serve as an early biomarker of PH.

## 1 Introduction

COPD is the second most common cause of pulmonary hypertension after left sided heart disease [1]. Pulmonary hypertension (Cor Pulmonale when associated with right heart failure in COPD) is relatively common in severe COPD and is present in >50% of patients awaiting lung transplantation [2]. The incidence increases with increasing severity of COPD, with pulmonary hypertension present in 5% of moderate (GOLD stage II), 27% of severe (GOLD stage III) and 53% of very severe (GOLD stage IV) COPD [3]. The true incidence in mild to moderate COPD however is poorly appreciated due to the absence of large scale epidemiological studies [4]. In comparison to other causes of pulmonary hypertension the pulmonary pressure elevations are modest, however survival is poor and correlates better with the pulmonary pressures and pulmonary vascular resistance than with the severity of airflow obstruction [5], [6], [7], [8].

Pulmonary hypertension is defined as a mean pulmonary arterial pressure (mPAP) greater than or equal to 25 mmHg at rest [9]. However: Pulmonary vascular changes occur in mild COPD before pulmonary hypertension occurs [10]; Right ventricular dysfunction has been observed in normoxaemic COPD patients with normal pulmonary arterial pressures [11]; and right ventricular hypertrophy is one of the earliest cardiac changes and is present even in mild COPD [12], [13], [14]. Additionally in both mice and guinea pig models, vascular remodelling occurs even before the onset of emphysema [15], [16], [17]. The notion of an elevated rest pressure as a cut-off is also restrictive in evaluating the role of the pulmonary arteries and right heart as even minimal exertion causes marked elevations in mPAP and oxygen desaturation in COPD, with exercise induced pulmonary hypertension present in 35%–58% of patients with normoxaemia or mild hypoxia [5], [18], [19], [20].

Therefore the pulmonary hypertension model, whereby right ventricular failure occurs once pulmonary hypertension exists, is a misnomer, and an alternate should be considered - the right ventriculo-arterial model.

### 1.1 Vascular changes

Vascular remodelling within COPD develops secondary to intimal hyperplasia, with muscularisation of the arterioles and with rather little change in the tunica media [21], [22]. This hyperplasia is induced by proliferation of smooth muscle cells and the deposition of elastic and collagen fibres [23], [24]. Originally hypoxia was held to be the driving force behind this remodelling, however similar arterial changes are present in smokers with and without COPD, and arterial changes can be observed in mild COPD when hypoxia is yet to develop [10], [23], [25]. The vascular remodelling in COPD is most likely due to endothelial dysfunction and apoptosis which in turn causes remodelling, capillary loss and small vessel thrombosis [26]. This is likely due to a combination of local inflammatory mediators released from the endothelial dysfunction in the adjacent pulmonary parenchyma, and direct effects from the inhaled cigarette smoke on the vascular endothelium [24], [26]. However an experimental study by Ferrer et al. in guinea pig models shows hypoxia still plays a contributory role, as a hypoxic environment after cigarette exposure induced significantly greater changes in pulmonary arterial pressures and pulmonary vascular remodelling than cigarette smoking or hypoxia alone [17]. More recently the pulmonary vascular sympathetic nerves which control the pulmopulmonary baroreceptor reflex originating at the pulmonary trunk has been proposed to be of significant importance in the development of pulmonary arterial hypertension [27]. This is evidenced by a rise in pulmonary arterial pressures and pulmonary vascular resistance (PVR) induced by stretching the branch pulmonary arteries [27], [28], [29], [30]. In a single study by Chen et al. [31] on 21 patients with significant pulmonary hypertension despite maximum medical therapy, intravascular pulmonary artery denervation with cessation of medical therapy resulted in significant improvement in pulmonary pressures and exercise capacity. However the role of this reflex in humans is still controversial and has also yet to be evaluated in COPD induced pulmonary hypertension [32].

#### 1.1.1 The right ventriculo-arterial model

##### 1.1.1.1 Right ventricle

The right ventricle (RV) got off to an inauspicious start in the 1940s and 50s when a series of papers showed no significant alterations in the pulmonary arterial or systemic pressures following open ablation of the right heart in a dog model, leading one of the authors to conclude that “a normal, contractile right ventricular wall is not necessary for the maintenance of a normal circulation” [33], [34], [35]. Despite this apparent swan song for a significant role of the right ventricle, a paper in 1982 demonstrated that the open pericardial model did not accurately represent the functional haemodynamics of the heart [36]. Goldstein et al. demonstrated that when the right coronary artery was infarcted within an intact pericardium there was a profound and rapid fall in left ventricular pressures due to a fall in left ventricular (LV) preload, equalisation of RV and LV pressures and elevated intrapericardial pressures which did not occur when they repeated their experiment after pericardiectomy. Thus there is a marked ventricular interdependence due to the shared pericardial compartment, shared interventricular septum and the dependence of the left heart preload on the output of the right [37], [38], [39], [40], [41], [42], [43].

Recent work has shown the importance of the right heart in health and disease. In health the right ventricle responds to exercise, obesity and traditional cardiovascular risk factors in a manner similar to, but independent of, the left ventricle [44], [45], [46]. A decreased RV mass is associated with an increased risk of developing dyspnoea, and an increased RV mass is associated with an increased risk of future congestive cardiac failure and cardiovascular mortality in a healthy population [47], [48]. In patients with COPD, right ventricular remodelling is apparent even early in the disease with right ventricular hypertrophy and diastolic dysfunction present in mild and moderate COPD with systolic impairment occurring in severe COPD [12], [13]. This has significant implications with right ventricular dysfunction associated with poorer exercise capabilities and increased mortality independent of pulmonary function tests [49], [50].

##### 1.1.1.2 Pulmonary artery

There has been a great deal of recent interest in systemic arterial stiffening in COPD due to the mechanistically plausible link this provides between COPD and the excess cardiovascular mortality associated with this condition [51]. In comparison to the systemic arterial circulation there are several key differences in the nature of the vascular tree between that of the pulmonary and systemic circulation. The pulmonary artery is a low pressure, high distensibility system which acts to transform the highly pulsatile right ventricular output into the near steady flow at the capillary level [52]. The distal pulmonary vascular bed is also comprised of highly distensible vessels that, through distension and recruitment of the microvascular bed, are able to accommodate large increases in volume such as during exercise. This response is the opposite of that experienced in the systemic circulation where exercise induces vasoconstriction in the majority of the vascular tree other than the vessels supplying the muscles. This inbuilt reserve means that resting pulmonary arterial pressures will only rise relatively late in a disease process when 60–70% of the bed is obstructed [53]. The peripheral distensability of the vessels also differs from the systemic circulation where only the proximal arteries act as capacitance vessels, expanding and contracting with each beat of the heart, while the peripheral vessels are responsible for resistance. Due to the dual function of the vessels, there is an inverse relationship between the resistance and capacitance of the pulmonary circulation, with the product of the two (the resistance-compliance time constant) remains the same in both health and disease [54].

The pulmonary arteries and microcirculation have distinct effects on the right ventricular afterload which can be split into two key components – the steady component required to drive and maintain forward flow and the oscillatory component required to overcome the pulsatile component of flow. The steady component is a function of peripheral vascular resistance whereas the oscillatory component is a function both proximal and distal pulmonary arterial stiffness [55]. Even in the healthy adult, 30% of the right ventricular output is spent on generating pulsations (compared with only 10% in the systemic circulation) [56]. This energy is considered wasted in terms of ventricular power output, but is crucial in acting as a blood reservoir and maintaining steady flow through the pulmonary tree [57]. As the pulmonary artery stiffens the oscillatory component of the workload increases, although remains as a constant of the total energy expenditure of the right ventricle [58], [59]. As the artery stiffens it also starts to dilate and become less compliant, and the dilation of this in relation to the aorta has been shown to be a useful clinical marker in COPD, with a PA:aortic ratio>1 an independent predictor of future acute exacerbations, exercise capacity, pulmonary hypertension and mortality [60], [61], [62], [63].

The compliance of the pulmonary artery is a key component in decoupling the right ventricle from the pulmonary bed, allowing the right ventricle to work at maximum efficiency and protecting the microcirculation from large pressure gradients [52], [64]. Indeed the stiffness of the pulmonary artery is a strong determinant of right ventricular function [65], and increased stiffness causes distal pulmonary arterial endothelial dysfunction and inflammation [66], [67]. Increased stiffness is independently associated with reduced functional capacity [68], and higher mortality than the pulmonary artery pressures or pulmonary vascular resistance [69], [70], [71], [72], [73]. While vascular distensability is partly dependant on underlying distending pressures [74], [75], multiple studies have shown the intrinsic stiffness of the arterial walls to be increased in pulmonary hypertension independently of these [76], [77], [78], [79], [80].

##### 1.1.1.3 Right ventriculo-arterial model

**Fig. 1:**
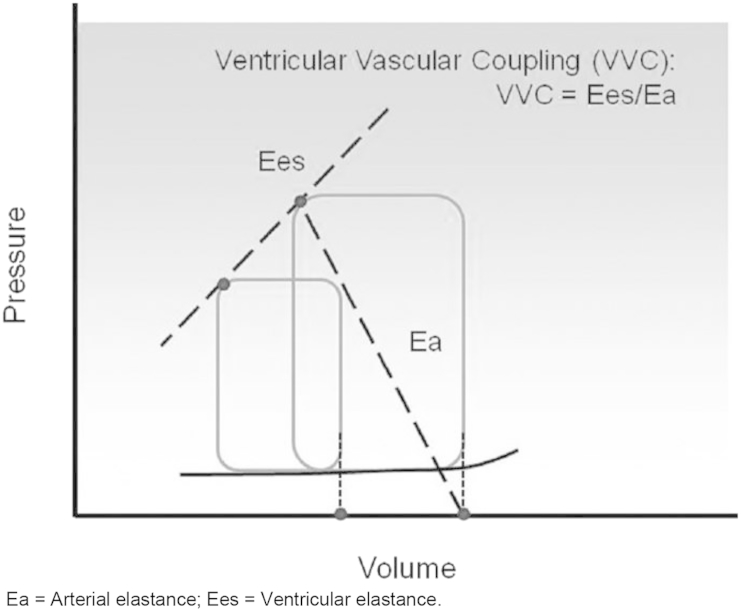
Calculation of E_A_:E_ES_ in the right ventricle and pulmonary artery. Ventricular end systolic pressure-volume is linear and characterised by the slope Ees and is generated by measuring pressure-volume loops under gradated preload and afterload conditions. Reproduced from Wang et al., 2011 [100]. Ea = Arterial elastance; Ees = Ventricular elastance.

Optimal ventriculo-arterial coupling occurs when there is maximal transference of potential energy from one elastic chamber to another, e.g. from the ventricle to the artery. This is dependent on the elastance – the change in pressure for a given change in volume – of the two chambers [81]. The ventricular elastance (E_ES_) is a load independent measure of the ventricular contractility combined with the modulating effects of the geometric and structural properties of the ventricle [82]. It relies on measuring the response of the ventricular pressure to increasing volumes (see Fig. 1). The arterial elastance (E_A_) is an index incorporating the elements affecting arterial load including pulmonary vascular resistance, arterial compliance, characteristic impedance, and systolic and diastolic time interval [83], [84]. The E_A_/E_ES_ calculates the extent of ventriculo-arterial coupling.

At rest the ventricle and pulmonary artery are coupled so that the right ventricle is working at optimal efficiency which occurs when the stroke work is maximal with minimal oxygen consumption [55], [85], [86], [87]. This has been shown to occur when E_A_ is half E_ES_ [87], [88], [89]. In exercise this moves towards optimal coupling when there is maximal cardiac efficiency which is where there is maximum flow generation with minimal energy loss (E_A_ = E_ES_) [90], [91], [92]. Interestingly the optimal values for energy efficiency and cardiac efficiency are similar throughout a variety of studied mammals suggesting it has been conserved throughout mammalian evolution [93], [94]. During exercise, arterial stiffness has a progressively and intensity-dependant greater impact on E_A_ than PVR with the result that E_A_ increases on exercise despite a fall in PVR in a manner paralleling the rise in arterial stiffness [95], [96], [97]. A similar relationship is present in pulmonary hypertension whereby pulmonary stiffness has a 1.2–18 fold greater contribution to right ventricular workload than PVR [74], [76], [54]. In the systemic circulation, age and hypertension (two known factors associated with increased arterial stiffness) result in an exaggerated increase in E_A_ during exercise increasing the pulsatile load on the heart and in turn decreasing left ventricular efficiency [90], [98]. This exaggerated response secondary to arterial stiffening may explain the rapid rise in mPAP observed in COPD during exercise despite a stable or minimal rise in PVR [18]. Indeed in a study by Hilde et al., an increase in pulmonary arterial stiffness was observed during exercise with a greater contribution from this to changes in mPAP than PVR [3]. Further indirect evidence comes from a study by Kubo et al. which showed the pulmonary arterial wall thickness to be related to exercise pulmonary pressures rather than resting pulmonary pressures, and to correlate highly with change in pressure from rest to exercise [99].

When the E_A_ increases so that the E_A_:E_ES_ is consistently above 0.5 the ventricle adapts to the increased workload by inducing hypertrophy [101]. This increases the E_ES_ thereby returning the ventriculo-arterial system to its most energy efficient state, albeit at a slightly higher energy consumption than previously [102], [103], [104]. As E_A_ continues to rise the right ventricle eventually fails to adapt with a resultant decrease in the E_ES_ and thereby a rapid increase in E_A_/E_ES_ indicating uncoupling and reduced myocardial efficiency [87], [105].

Fourie et al. demonstrated that myocardial efficiency reached a maximum when the E_A_ reached 1mmHgml^−1^ (corresponding to an mPAP of 20 mmHg), while maximum stroke work corresponded to an E_A_ of 2 mmHgml^−1^ (corresponding to a MPAP of 30–40 mmHg) [52]. This corresponds with observations that at approximately 40 mmHg the pulmonary artery reaches it maximum dimension and minimal distensability [78], [105], [106]. Consistent with this, Stevens et al. demonstrated that in patients with pulmonary hypertension there was a curvilinear relationship between RV function and PA distensibility with marked loss of pulmonary artery distensibility without commensurate loss of ventricular function until only minimal distensibility remained when a rapid decompensation of the right ventricle occurred [76]. A similar curvilinear relationship has also been observed in COPD between pulmonary arterial stiffness and peripheral vascular resistance, with a rapid rise in PVR only occurring after significant stiffening of the pulmonary arteries has occurred [11]. Pulmonary stiffness is increased early in pulmonary hypertension development, and is increased even in those with exercise induced pulmonary hypertension, both in mixed pulmonary arterial hypertension studies and in COPD [3], [11], [78], [106]. This combination of features suggest pulmonary arterial stiffness as a promising biomarker for detection of early disease and as a potential therapeutic target before end stage arterial remodelling occurs with dire consequences for the right ventricle.

#### 1.1.2 Measuring pulmonary artery stiffness

The stiffness of the pulmonary artery can be quantified using a variety of metrics, with these measuring stiffness locally, regionally or systemically. Table 1 summarises the various methods of measuring arterial stiffness and the common methods for acquiring these. As can be seen from the table the majority of these require knowledge of the arterial pressures to calculate the stiffness. In the arterial circulation this is not a significant issue as the brachial arterial pressures are readily obtained with a sphygmomanometer, as mean BP and diastolic BP are relatively constant throughout the large arteries [107]. However in the pulmonary arteries this provides a significant hurdle as an external measurement of the pressures is not readily available. Despite numerous attempts to quantify pulmonary arterial pressures and vascular resistance using echocardiography, CT and MRI, none of these have yet proven accurate enough to negate the need for invasive measurement with right heart catheterisation [108], [109], [110], [111], [112], [113], [114], [115].

##### 1.1.2.1 Right heart catheterisation

Right heart catheterisation can be used to calculate several metrics of arterial stiffness, including capacitance, PWV and elastance, although the latter two require dedicated catheters and equipment not routinely available in the catheter laboratory. In addition, it can be combined with techniques for the visualisation of the pulmonary artery cross sectional area such as MRI or intravascular ultrasound, to derive a wide range of metrics of arterial stiffness and ventriculo-arterial coupling. Table 2 summarises the studies looking at invasive assessment of pulmonary arterial stiffness. Despite its utility, RHC has numerous disincentives including its invasive nature, cost, and exposure to ionising radiation. While the combination of MRI and right heart catheterisation allows catheter guidance without exposure to ionising radiation and simultaneous acquisition of anatomical and pressure measurements, it does not negate the invasive or costly nature of the study [116], [117]. Of the measures of arterial stiffness, only pulsatility and pulse wave velocity can be derived entirely non-invasively.

##### 1.1.2.2 Pulsatility

Multiple studies have shown reduced pulsatility in pulmonary hypertension, [68], [69], [80], [106], [123], [124], [125]. A single echocardiography study has shown reduced pulmonary artery pulsatility in COPD, which correlated with right ventricular functional parameters [126]. A single abstract using cardiac MR has also described decreasing pulsatility with increasing severity of COPD [127] (Table 3). However, as discussed above, the pulmonary stiffness is dependant not only upon distending pressures, but also upon the intrinsic elasticity of the pulmonary vessel wall, thus a fall in pulmonary pulsatility does not differentiate a fall in pulsatility due to a fall in stroke volume or in distending pressures from that of a rise in arterial stiffness.

##### 1.1.2.3 Pulse wave velocity

Pulse wave velocity (PWV) is the speed at which the pressure wave, generated by ventricular contraction, is transmitted through the arterial tree. This is directly related to vessel wall elasticity, with increased stiffness leading to increased PWV [128]. A reflected pressure wave is present which is generated when the pressure wave is reflected from the termination of the arteries, with alterations in the smallest arteries increasing the size of the reflected wave while large artery stiffening increases the PWV [118]. In health this reflected wave only represents a tiny fraction of the total output energy, and arrives in diastole and reduces preload of the subsequent ventricular contraction [92], [118], [129], [130]. However in pulmonary hypertension this can reach 30% of the forward wave energy. with an increased PWV resulting in the earlier arrival of the reflected wave during systole thereby increasing the afterload of the heart and thus cardiac workload [77], [119], [122], [131], [132], [133].

**Fig. 2:**
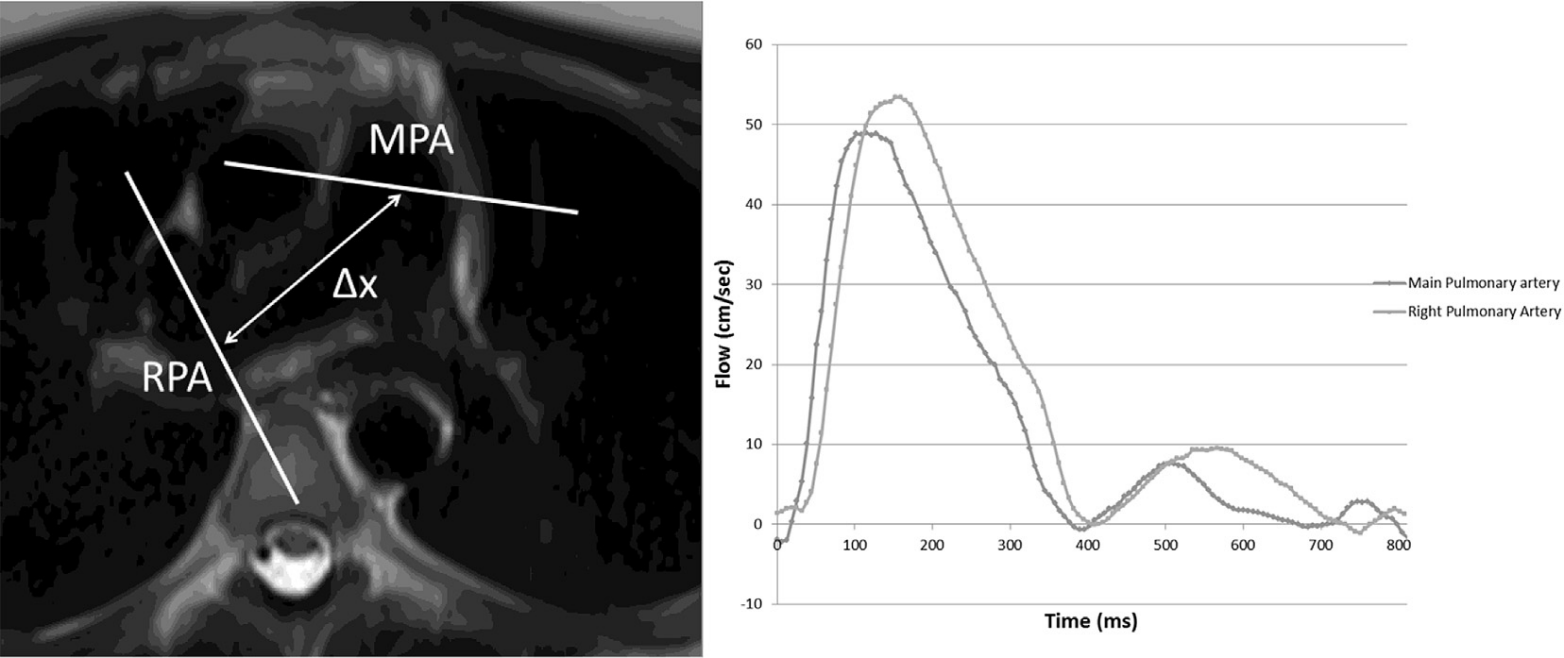
Measurement of PWV using MRI: The location of the two phase contrast planes are delineated on the left image with the distance between them measured. The two separate waveforms are traced on the right relative to the triggering R-wave used to start image acquisition. The time between the waves arriving at the two consecutive locations can then be measured allowing calculation of the speed of the pulse wave.

PWV is an established marker of arterial stiffness in the systemic circulation with aortic pulse wave velocity shown to be associated with future cardiovascular events, and to improve risk stratification [134]. Transcutaneous waveform analysis methods are the gold standard for aortic PWV measurement including pressure [135], distension [136], and flow waveform [137]. However a transcutaneous approach is not possible for the pulmonary arteries. While PWV can be measured invasively on right heart catheterisation [118], magnetic resonance imaging is a possible alternative for non-invasive assessment of the pulmonary arteries using flow waveforms [138]. It is performed by the acquisition of ECG gated phase contrast sequences which measure flow the in proximal main pulmonary artery and in the mid right or left pulmonary arteries. A distance can be calculated between the two imaging planes (Fig. 2), and the pulse wave timed from when it leaves the main pulmonary artery until it arrives in branch pulmonary arteries thus allowing a speed between the two points to be generated [138]. MRI derived pulse wave velocity has been previously validated against both invasive and non-invasive techniques in the aorta [139], [140], [141]. Several studies have shown this to be a feasible and reproducible technique in the pulmonary arteries in healthy volunteers [138], [142], with the observed pulse wave velocity of 1.96–2.3 ms^−^^1^ similar to that obtained invasively [118], [129]. Pulse wave velocity is raised in pulmonary hypertension on both invasive and MRI assessment (See Table 2) [121], [143]. To date there have been no studies assessing pulmonary PWV in COPD.

#### 1.1.3 Pulmonary arterial stiffness as a treatment target

Traditional treatments of pulmonary hypertension such as phosphodiesterase inhibitors and endothelin-1 receptor antagonists have yielded disappointing results despite their success in treating pulmonary hypertension, primarily due to worsening ventilatory-perfusion mismatches [144], [145], [146], [147], [148]. However these treatments have all focussed on altering the peripheral vasculature. Given the greater impact of pulmonary stiffness on remodelling, drugs to target and ameliorate this process may be a novel route to target. Due to the relatively recent identification of the importance of pulmonary arterial stiffness in right ventricular remodelling, there have been relatively few studies targeted at its treatment. Despite this there are several studies suggestive of promising avenues for further exploration.

Statins have been proposed as a route to reduce systemic arterial stiffness [149], although a meta-analysis showed mixed response with two studies showing improvement, one showing no change and another showing progression of stiffening [150]. Statins in guinea pigs and mice with emphysema have been shown to reverse arterial remodelling and halt the formation of emphysema [151], [152]. Indirect evidence of the benefits from statins in COPD related pulmonary hypertension come from observational studies showing lower pulmonary arterial pressures in patients on statins prior to right heart catheterisation [153]. Furthermore a study on pulmonary arterial stiffness showed that other than mPAP, the only other factors independently associated with pulmonary arterial stiffness was LDL cholesterol and BMI [121]. Statins are also associated with increased exercise capacity and reduced mortality in COPD [154], [155], [156], however due to the observational nature of these studies the underpinning mechanisms are still in debate [157], [158].

Xanthine oxidase inhibitors have been shown to improve endothelial function and arterial stiffness in the systemic circulation [163], [164], [165], [166], [167]. They have also been shown to reduce endothelial dysfunction induced by smoking and obstructive sleep apnoea [168], [169], [170] – a condition similar to COPD in its effects of intermittent hypoxia [171]. Additionally allopurinol inhibits hypoxia induced pulmonary vasoconstriction, pulmonary hypertension, endothelial dysfunction and vascular remodelling in hypoxic animal models of pulmonary hypertension [172], [173], [174], [175], [176]. It has been shown to reduce airway reactive nitrogen species production in COPD, however its effects on smoking induced pulmonary vascular damage has not been assessed [177].

As with xanthine oxidase inhibitors, mineralocorticoid receptor antagonists have been shown to improve systemic endothelial function and arterial stiffness [178], [179], [180]. In 2 separate mouse models of pulmonary hypertension, mineralocorticoid receptor antagonists prevented or reversed adverse pulmonary vascular remodelling with improved pulmonary vascular resistance, pulmonary artery pressure, and remodelling of the right ventricle [181]. A group in Bethesda, USA are currently recruiting patients with pulmonary hypertension without right heart failure to evaluate its efficacy in humans in modulating pulmonary vascular remodelling [182]. Its use in COPD has not currently been assessed.

Long term oxygen therapy improves survival in severe COPD and reduces pulmonary arterial pressures and vascular resistance while improving cardiac output [6], [183]. It has also been shown to reduce peripheral arterial stiffness in COPD, however its effects on the pulmonary arterial stiffness has not been evaluated [184].

There are also several new classes of pharmaceutical agents currently being designed which specifically target the processes causing arterial stiffening. Currently these are all being targeted at systemic vascular remodelling and are in the pre-clinical stages, however should they prove successful at preventing or reversing systemic arterial remodelling they would be promising agents for evaluation in pulmonary remodelling. The most promising of these are TGF-B1 antagonists, and drugs targeting the advanced glycation end products (AGEs) [185]. TGF-B1 is responsible for extracellular matrix remodelling and vascular fibrosis in chronic inflammatory conditions, whilst AGE form irreversible cross-links with collagen and elastin, reducing the elasticity of the arterial wall [185].

## 2 Conclusions

Pulmonary arterial stiffness holds a crucial role in right ventricular dysfunction in pulmonary hypertension, and demonstrates promise as a useful biomarker in the development of this disease. Early work shows similar promise in COPD, and further work is warranted to explore this promising new area.

## Figures and Tables

**Table 1:** Measures of arterial stiffness – Calculations, definitions and alternate means of assessment.

	Calculation	Definition	Method of assessment
Local			
Pulsatility (%)	(maxA – minA)/minA X 100	Relative change in lumen area during the cardiac cycle	MRI Echocardiography IVUS
Compliance (mm^2^/mmHg)	(maxA – minA)/PP	Absolute change in lumen area for a given change in pressure	RHC plus MRI/Echo/IVUS
Distensibility (%/mmHg)	[(maxA – minA)/PP X minA] X 100	Relative change in lumen area for a given change in pressure	RHC plus MRI/Echo/IVUS
Elastic modulus (mmHg)	PP X minA/(maxA – minA)	Pressure change driving a relative increase in lumen area	RHC plus MRI/Echo/IVUS
Stiffness index (N/A)	Ln(sPAP/dPAP)/[(maxA – minA)/minA]	Slope of the function between distending arterial pressure and arterial distension	RHC plus MRI/Echo/IVUS
Young's elastic modulus	[3(1 + minA/WCSA)]/Distensibility	Wall thickness for a given distensibility	RHC plus IVUS
Regional			
Capacitance (mm^3^/mmHg)	SV/PP	Change in volume associated with a given change in pressure	RHC
PWV (ms^−1^)	Δd/Δt (TT technique) ΔQ/ΔA (Flow-area technique)	Speed of transmission of pressure wave	RHC MRI
Systemic			
Elastance (mmHgml^−1^)	E_A_/E_ES_ = (ESP/SV)/(ESP/ESV-V0)	Change in pressure for a given change in volume	RHC with flow-volume loops

A = area; d = distance; dPAP = diastolic pulmonary artery pressure; EA = Arterial elastance; Echo = echocardiography; EES = Ventricular elastance; IVUS = intravascular ultrasound; maxA = maximum cross sectional area; mina = minimum cross sectional area; MRI = magnetic resonance imaging; PP = pulse pressure; PWV = pulse wave velocity; Q = Flow over a single heart beat; RHC = right heart catheter; sPAP = systolic pulmonary artery pressure; SV = stroke volume; t = time; WCSA = Wall cross sectional area.

**Table 2:** Summary of studies looking at invasive measurement of pulmonary arterial stiffness.

Study	Population	No	Method	Finding
Milnor et al. [118]	Mixed PH	7	RHC	Increased impedance in PH
Nakayama et al. [119]	Mixed PH	62	RHC	Increased augmentation index and earlier inflection time in PH
Castelain et al. [77]	Mixed PH	14	RHC	Increased augmentation index and earlier inflection time in PH
Mahapatra et al. [70]	Mixed PH	104	RHC	Capacitance strongest predictor of mortality in PH. On multivariate analysis, capacitance was the only statistically significant predictor of mortality.
Hilde et al. [11]^a^	COPD	98	RHC	PA compliance greatly reduced in COPD Gold 2,3 and 4
Hilde at al [3]^a^	COPD	98	RHC	PA compliance fell with exercise in patients with and without PH, with similar elevations in mPAP during exercise between the two groups.
Muthurangu et al. [120]	IPAH	17	RHC with MRI	Inverse relationship between compliance and PVRI and mPAP. A fall in compliance was evident in 7/17 following nitric oxide inhalation
Kuehne et al. [87]	Mixed	12	RHC with MRI	Increased elastance in PH, with resultant ventriculo-arterial uncoupling
Kopec et al. [121]	IPAH	26	RHC with IVUS	PWV high in IPAH (mean PWV 10 ms^−^^1^) with excellent correlation with compliance (β = −0.81) and reasonable correlation with mPAP (β = 0.48)
Lau et al., 2012 [79]	IPAH	8	RHC with IVUS	Increase in all measure of pulmonary arterial stiffness. Inverse curvilinear relationship between mPAP and compliance and distensibility. No change in PA stiffness following 6 months of bosentan therapy.
Rodes-Cabau et al. [72]	IPAH	20	RHC with IVUS	Reduced pulsatility in PH, with reduced pulsatility predictive of future mortality. Epoprostenol infusion increased pulsatility by 53%.
Lau et al., 2014 [122]	IPAH	5	RHC with IVUS	High PWV in IPAH (mean PWV 10.6 ms^−1^) with the reflected backward compression wave carrying 31% of the energy of the forward compression wave

COPD = chronic obstructive pulmonary disease; CoV = Coefficient of variation; EIPH = Exercise induced pulmonary hypertension; FEV1 = Forced expiratory volume in 1 s; HV = Healthy volunteers; IPAH = Idiopathic pulmonary arterial hypertension; NYHA = New York heart association; PASP = Pulmonary artery systolic pressure; PH = Pulmonary hypertension; PWV = pulse wave velocity; QA = flow by area; TT = transition time; 6MWT = 6 min walking test.

a Drawn from the same population.

**Table 3:** Summary of studies looking at non-invasive measurement of pulmonary arterial stiffness.

Study	Population	No	Method	Finding
Pulsatility				
Bogren et al. [159]^b^	Mixed PH	4	MRI	Pulsatility 23% in HV and 8% in PH
Paz et al. [160]	HV	9	MRI	No difference in pulsatility between the main pulmonary artery, right pulmonary artery and left pulmonary arteries.
Gan et al. [69]	Suspected PH	70	MRI	Right pulmonary artery pulsatility showed a curvilinear relationship with mortality. Pulsatility <16%
Jardim et al. [125]^b^	IPAH	19	MRI	<10% pulsatility predicts non-responders to acute vasodilator testing with 100% sens and 56% spec.
Sanz et al. [124]^a^	Suspected PH	59	MRI	Pulsatility 41% in non-PH and 17.4% in PH. No difference in pulsatility between different causes of PH.
Sanz et al. [78]^a^	Suspected PH	94	MRI	No difference in pulsatility between group without PH and EIPH. Pulsatility <40% predicted PH with 93% sens. and 63% spec.
Kang et al. [68]	Mixed PH	35	MRI	Pulsatility correlated with 6MWT (R2 = 0.61, p < 0.001)<20% pulsatility predicted poor function (6MWT <400m) with 82% sens. and 94% spec.
Stevens et al. [76]^a^	Suspected PH	124	MRI	Pulsatility correlates with right ventricular function
Stevens et al. [161]	Suspected PH	43	MRI	Pulsatility correlated with exercise capacity while RHC pressures did not.
Swift et al. [106]	Suspected PH	134	MRI	Pulsatility elevated even in very mild elevations in PVR. Pulsatility predicted mortality.
Revel et al. [162]^b^	Suspected PH	45	CT	Pulsatility of 16.5% predicted PH with sens 86% and spec 96%.
Pasierski et al. [80]	Suspected PH	19	Echo	Pulsatility reduced in pulmonary hypertension with a linear relationship between pulsatility and PASP
Mahapatra et al. [71]	Suspected PH	54	Echo	Capacitance was the strongest predictor of mortality including invasive pressure measurements
Ertan et al. [126]	COPD	54	Echo	Right pulmonary artery pulsatility reduced in COPD patients, with significant differences between NYHA functional classes.
Liu et al. [127]	COPD	135	MRI	Pulsatility falls with increasing severity of COPD, with a positive association with %predicted FEV1 and inversely correlated with %emphysema
PWV				
Peng et al. [142]	HV	17	MRI	PWV 1.96 ± 0.27 with high intra-scan and inter-scan reproducibility (5.5% and −10.9% respectively)
Bradlow et al. [138]	HV	10	MRI	No significant difference in PWV using the left or right pulmonary arteries. CoV 12% for both intra and inter-observer assessment.
Ibrahim et al. [143]	Heterogeneous	33	MRI	PWV raised in pulmonary hypertension. Comparable measurements between TT and QA methods.

COPD = chronic obstructive pulmonary disease; CoV = Coefficient of variation; EIPH = Exercise induced pulmonary hypertension; FEV1 = Forced expiratory volume in 1 s; HV = Healthy volunteers; IPAH = Idiopathic pulmonary arterial hypertension; NYHA = New York heart association; PASP = Pulmonary artery systolic pressure; PH = Pulmonary hypertension; PWV = pulse wave velocity; QA = flow by area; TT = transition time; 6MWT = 6 min walking test.

a Drawn from the same population.

b Different calculation for % pulsatility so not directly comparable to other studies.
